# Therapeutic Options for Systemic Sclerosis: Current and Future Perspectives in Tackling Immune-Mediated Fibrosis

**DOI:** 10.3390/biomedicines10020316

**Published:** 2022-01-29

**Authors:** Theodoros-Ioannis Papadimitriou, Arjan van Caam, Peter M. van der Kraan, Rogier M. Thurlings

**Affiliations:** Department of Rheumatic Diseases, Radboud University Medical Center, 6525 GA Nijmegen, The Netherlands; Arjan.vanCaam@radboudumc.nl (A.v.C.); Peter.vanderKraan@radboudumc.nl (P.M.v.d.K.); rogier.thurlings@radboudumc.nl (R.M.T.)

**Keywords:** systemic sclerosis, immune cells, anti-inflammatory, therapy, fibrosis

## Abstract

Systemic sclerosis (SSc) is a severe auto-immune, rheumatic disease, characterized by excessive fibrosis of the skin and visceral organs. SSc is accompanied by high morbidity and mortality rates, and unfortunately, few disease-modifying therapies are currently available. Inflammation, vasculopathy, and fibrosis are the key hallmarks of SSc pathology. In this narrative review, we examine the relationship between inflammation and fibrosis and provide an overview of the efficacy of current and novel treatment options in diminishing SSc-related fibrosis based on selected clinical trials. To do this, we first discuss inflammatory pathways of both the innate and acquired immune systems that are associated with SSc pathophysiology. Secondly, we review evidence supporting the use of first-line therapies in SSc patients. In addition, T cell-, B cell-, and cytokine-specific treatments that have been utilized in SSc are explored. Finally, the potential effectiveness of tyrosine kinase inhibitors and other novel therapeutic approaches in reducing fibrosis is highlighted.

## 1. Introduction

Systemic sclerosis (SSc) is a severe rheumatic, auto-immune disease which affects up to 20 people per 100,000 and women up to nine times more often than men [1,2,3]. Inflammation, vasculopathy, and fibrosis are the key hallmarks of SSc. Patients suffer from fibrotic skin lesions, and as the disease progresses, the function of internal organs including the heart, lungs, gastrointestinal tract, and kidneys deteriorates due to fibrosis. The disease has a high morbidity and greatly negatively affects the quality of life and life expectancy of patients.

**Figure 1:**
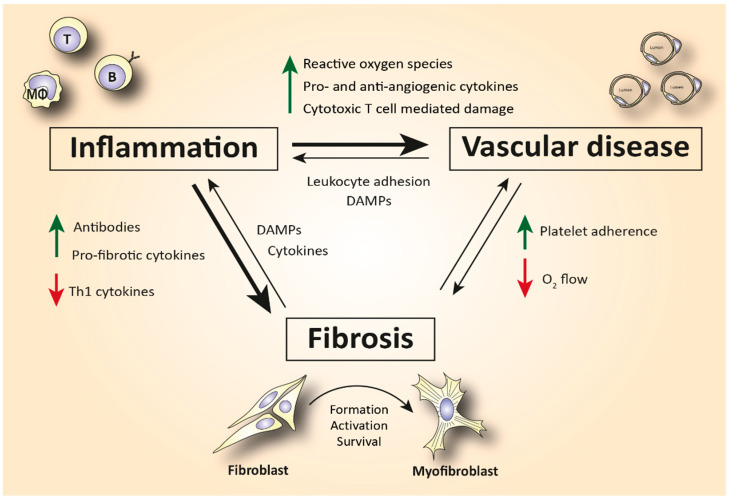
Simplified schematic representation of the complex interplay between inflammation, vasculopathy, and fibrosis in SSc. Abbreviations: Th1 = T helper type 1 cells; DAMPs = damage-associated molecular patterns; T = T lymphocyte; B = B lymphocyte; MΦ = macrophage.

Although the pathophysiology of SSc has not been elucidated yet, the immune system has been hypothesized as an important driver of the disease (Figure 1). SSc patients exhibit disease-specific autoantibodies, and a typical cytokine profile in their blood indicating immune cell activation. This profile is characterized by increased T helper 2 (Th2) and decreased T helper 1 (Th1) cytokines [4,5]. Furthermore, in skin biopsies of early disease patients, perivascular accumulation of immune cells, such as CD4+ and CD8+ cytotoxic T cells (CTLs), can be observed [6]. This immune activation can cause (or is maybe a response to) capillary damage, which leads to capillary breakdown, adherence of platelets, and activation of pro-fibrotic pathways. Additionally, vascular injury causes damage and apoptosis of endothelial cells. The release of internal damage-associated molecular patterns (DAMPs) increases microvascular permeability which causes additional recruitment of immune cells to the endothelium and therefore increased immune cell activation and inflammation [7].

Activation of the immune system is also linked to fibrosis. For example, Th2 cytokines, such as interleukin (IL)-4 and -13, can activate myofibroblasts [8]. Myofibroblasts are a strongly pro-fibrotic cell type which produces large amounts of extracellular matrix (ECM) molecules and matrix-strengthening enzymes. Furthermore, these cells also excrete growth factors and cytokines that worsen inflammation [9]. IL-4, IL-6, and transforming growth factor (TGF)-β are the predominant fibrogenic cytokines that cause subendothelial accumulation of fibrous tissue, leading to aberrant vascular remodeling [10]. In turn, this aberrant vascular remodeling makes the capillaries more prone to damage, fueling an immune response. 

The important role of the immune system in the pathogenesis of SSc is further supported by the fact that autologous hematopoietic stem cell transplantation (ASCT) can induce long-term disease remission in patients with SSc. However, ASCT is accompanied by a high mortality rate (~10%) and is only administered in the most severe cases of SSc [11]. Currently, there is no specific and effective disease-modifying treatment available, which, regarding the severity of the disease, results in an unmet medical need. Presently, mainly broad-spectrum immunosuppressive and anti-inflammatory drugs, developed for other auto-immune diseases, are used to treat SSc. In this narrative review, we discuss the effectiveness of the currently used and/or investigated agents in diminishing skin and lung fibrosis in scleroderma patients. For this, we first provide an overview of the main pathogenic pathways that are implicated in the disease. Secondly, drugs targeting these pathways are assessed for their treatment efficacy based on results from selected clinical trials. To write this review, the databases PubMed and clinicaltrials.gov were used to search for publications up to November 2021. Combinations of Medical Subject Headings (MeSH) terms referring to SSc (“Systemic sclerosis” OR “Scleroderma, Systemic”) and drugs of interest, e.g., “methotrexate”, were included in the search strategy.

## 2. The Role of Immune Cells in SSc-Related Inflammation and Fibrosis

A large body of evidence suggests that both the innate and the adaptive immune system are involved in the fibrogenesis of SSc.

### 2.1. The Role of Innate Immunity in SSc

The innate immune system mediates the immediate defensive response against pathogens and chemical or mechanical damage. An increased number of macrophages, mast cells, type 2 innate-like lymphoid cells, eosinophils, and plasmacytoid dendritic cells have been identified in SSc tissues [12]. The induction of excessive numbers of myofibroblasts that is observed in SSc is partly mediated by the previously mentioned innate immune cells. Other innate immune cell types such as platelets, neutrophils, and natural killer cells have also been found to be dysregulated in SSc pathology. However, only a few studies highlighting their role in SSc have been published. Thus, in this review, we focus on the cells of the innate immune system that are well characterized in SSc.

#### 2.1.1. Macrophages

**Figure 2:**
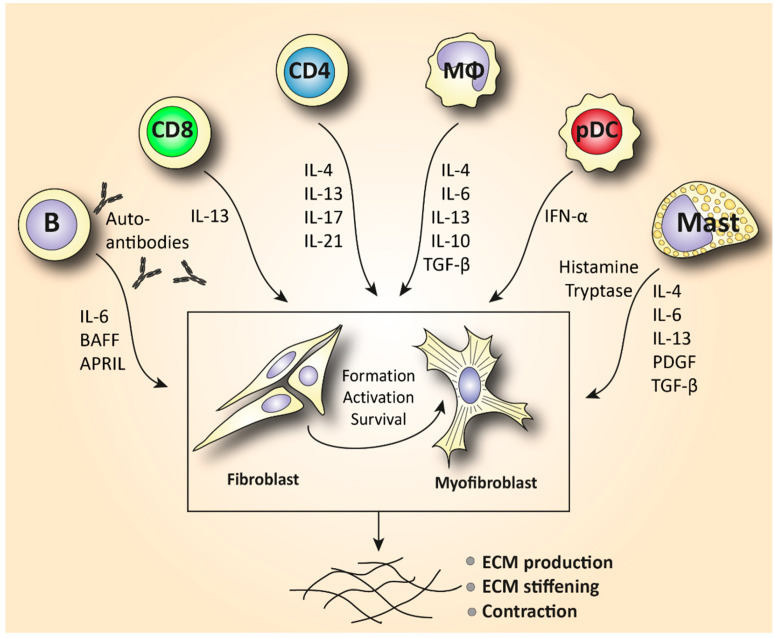
An overview of the main pathogenic mechanisms by which T cells, B cells, macrophages, mast cells, and plasmacytoid dendritic cells may contribute to (pro-)fibrotic manifestations in SSc immunopathology. Abbreviations: MΦ = macrophages; B = B lymphocyte; CD8 = cytotoxic T cell; CD4 = T helper cell; pDC = plasmacytoid dendritic cell; TGF-β = transforming growth factor-β; BAFF = B cell activating factor; APRIL = a proliferation-inducing ligand; PDGF = platelet-derived growth factor; ECM = extracellular matrix.

Macrophages are effector phagocytotic cells specialized in eliminating pathogens. These cells play an essential role in homeostatic manifestations related to the disposal of internal waste products and tissue repair. Monocyte-derived macrophages can be found in the blood circulation under inflammatory conditions but are prevalent in all human tissues as tissue-resident macrophages. However, macrophages exhibit a highly heterogeneous phenotype that is regulated by their microenvironment. Under the influence of certain inflammatory mediators, macrophages can be polarized in vitro towards a classically activated (M1) inflammatory phenotype or an alternatively activated (M2) tissue repair phenotype. M2 macrophages are often characterized by the secretion of large amounts of IL-10, TGF-β, and other pro-fibrotic cytokines such as IL-4, IL-6, and IL-13 that play an essential role in wound healing and tissue repair (Figure 2). These cytokines are also known to induce fibrosis by activating myofibroblasts [13]. On the other hand, in states of chronic tissue repair, M2 macrophages can also exhibit anti-fibrotic functions that lower the progression of fibrosis by suppressing local T helper cell responses and decreasing the production of ECM by (myo)fibroblasts [14,15]. Furthermore, to a certain extent, M1 and M2 phenotypes are artificial in vitro cell states. In vivo, different types of anti-inflammatory and anti-fibrotic macrophages have been reported in the process of wound healing, repair, and fibrosis [16].

In SSc, transcriptomic and immunohistochemistry studies identified a prominent pro-fibrotic M2 macrophage signature in patients’ skin and lung lesions that was correlated with an increased skin score and disease severity [17,18,19]. In addition, a dominant M2 monocyte signature has also been observed in SSc blood [20]. This notion suggests a role of M2 macrophages in the immunopathogenesis of SSc. The importance of macrophages in regulating fibrosis is further supported by the observation that mice lacking macrophages (with the use of liposomal chlodronate) exhibit reduced bleomycin-induced lung fibrosis [21]. In skin lesions, M2 macrophages are characterized by overexpression of the scavenger receptor CD163 or CD204 [22]. The number of CD163+ or CD204+ cells was found to be significantly expanded in SSc compared to healthy skin [23]. Strikingly, these activated macrophages were localized not only in the perivascular areas, but also between thickened collagen fibers. This indicates a potential role of tissue-infiltrating macrophages in the development of fibrosis [24]. However, it is worth mentioning that bone marrow-derived mesenchymal progenitors such as monocytes and fibrocytes may also be implicated in SSc-related fibrosis. These cells are able to migrate from the circulation to affected tissues such as the lungs, where they differentiae into activated (myo)fibroblasts [25]. Of note, it has been reported that fibrocytes were only detected in patients with connective tissue disorders (including SSc) and not in healthy donors [26]. In SSc, the frequency of circulating fibrocytes was positively correlated with an increased dermal thickness [27]. Furthermore, in a very recent single-cell RNA sequencing (sc-RNA seq) study, highly proliferating macrophages were only found in SSc and not healthy skin and were correlated with increased skin fibrosis [28]. All in all, the role of macrophages in fibrotic activation in SSc is prominent, but the signaling pathways characterizing their aberrant activation remain poorly understood. In addition, their role as regulators of the disease, in combination with deciphering a potential role of anti-fibrotic macrophages in SSc, definitely warrants further research. Unraveling the pathogenic mechanisms by which macrophages mediate fibrosis will likely contribute to targeted therapies that reduce fibrosis and ameliorate inflammation in SSc and other connective tissue disorders. To this end, future therapeutic options should opt to reduce the activation/number of activated pro-fibrotic and pro-inflammatory macrophages while boosting the activity of the anti-inflammatory and anti-fibrotic ones.

#### 2.1.2. Eosinophils and Mast Cells

As we discussed previously, serum levels of the cytokines IL-4, IL-10, and IL-13 are elevated in SSc patients and are correlated with increased fibrosis and disease severity. Meanwhile, IL-4 and IL-13 play an essential role in eosinophil-mediated inflammation, suggesting a potential role of eosinophils in SSc pathology [29,30]. Eosinophils are granulocytes derived from the myeloid stem cells that are part of the innate immune system. Elevated levels of eosinophils have been described in SSc and other diseases of the connective tissue [31]. In the peripheral blood of early untreated SSc patients, eosinophil counts were higher compared to patients with other major collagen diseases such as dermatomyositis, Sjogren’s syndrome, and systemic lupus erythematosus. Furthermore, eosinophil counts were significantly correlated with severe interstitial lung disease (ILD) and an increased skin thickness in patients with SSc, but not in patients with other collagen diseases [32]. In addition, the presence of skin ulcers in SSc has been associated with elevated counts of peripheral blood eosinophils [33]. Collectively, these results implicate eosinophils in the pathogenesis of SSc including the hallmarks of inflammation and vascular dysfunction. However, studies exploring the exact mechanism of inflammation or ulcer formation are lacking. 

Another cell type of the innate immune system derived from myeloid stem cells is mast cells. Mast cells have been detected in SSc tissues and can exhibit pro-fibrotic manifestations. Elevated infiltration of mast cells has been found in various SSc tissues including the skin and salivary glands [34,35,36]. Mast cell infiltration has been correlated with more severe disease phenotypes. Except for their well-established role in immediate inflammatory and allergic reactions, these cells are also implicated in cardiac, renal, and pulmonary fibrosis. Mast cells have been linked to fibrosis by their ability to produce pro-fibrotic cytokines such as IL-4, IL-6, IL-13, tumor necrosis factor alpha (TNF-α), platelet-derived growth factor (PDGF), and TGF-β [37]. This way, mast cells stimulate the production and activity of myofibroblasts (Figure 2). 

Serotonin is another molecule that is produced by mast cells. Serotonin can directly increase ECM deposition in primary skin fibroblasts in a TGF-β-dependent manner [38]. In addition, mast cells produce tryptase, a serine proteinase which triggers fibroblast proliferation and collagen production [39]. It was also demonstrated that fibroblast proliferation in patients’ lungs can be caused by mast cell-related histamine release [40]. More specifically, human primary lung fibroblasts that were cultured in the presence of physiologically relevant concentrations of histamine exhibited increased cell proliferation that was mediated through an H2 histamine receptor on the fibroblasts. Interestingly, the observed fibroblast proliferation was inhibited by an H2 antagonist, empowering the role of histamine release in fibroblast proliferation. Of note, mast cell depletion with phototherapy ameliorates SSc fibrosis in vivo [41]. Thus, targeting mast cells in systemic sclerosis might be an effective treatment approach. We believe that exploring the immunopathogenic role of eosinophils and mast cells with the currently available novel arsenal of biomolecular techniques will unveil new innate pathogenic pathways that could pave the way for novel treatment approaches.

#### 2.1.3. New Players: Innate Lymphoid Cells and Plasmacytoid Dendritic Cells

Among the family of innate immune cells, a highly interesting tissue-resident cell type that was recently shown to be involved in SSc is innate lymphoid cells (ILCs). These cells are derived from common lymphoid progenitors such as adaptive immune cells, but they lack rearranged antigen receptors. Therefore, ILCs do not exhibit antigen-specific responses but are characterized by a functional diversity similar to that of T lymphocytes. These cells have been categorized into distinct subtypes based on their cytokine and transcriptome profile. One of these subtypes is ILC2, which is identified by the expression of the transcription factor GATA-3. As with Th2 cells, ILC2s predominantly produce IL-4, IL-5, and IL-13 [42].

Intriguingly, locally accumulating ILC2s have emerged as a pivotal source of pro-fibrotic cytokines in inflammatory and fibrotic diseases [43]. Studies using the carbontetrachloride (CCl4)-induced liver fibrosis mouse model showed that ILC2s mediate liver fibrosis by producing the pro-fibrotic cytokine IL-13 [44]. This observation suggests that ILC2s may stimulate the activation of fibroblasts and thus increase tissue fibrosis. Indeed, ILC2 counts are significantly increased in both the skin and peripheral blood from patients with SSc compared to healthy controls, and their numbers are correlated with an increased skin thickness [45,46]. This notion is further supported by studies that have shown an elevated expression of the ILC2 cytokines IL-25, IL-33, and thymic stromal lymphopoietin (TSLP) in both the serum and skin of patients with SSc [46,47,48]. From a mechanistic point of view, TGF-β and IL-10 are two cytokines that have been implicated in ILC2-mediated skin fibrosis in SSc. Elevated TGF-β enhanced the in vitro pro-fibrotic function of ILC2s by increasing the activation of myofibroblasts and downregulating the levels of IL-10. Downregulation of IL-10 increased the production of collagen by dermal fibroblasts. In the same study, TGF-β inhibition combined with IL-10 administration prevented fibrotic manifestations in a mouse model recapitulating SSc [49]. Although there is a limited number of studies associating ILC2s in the pathogenesis of SSc, we believe that findings implicating TGF-β in their potential fibrotic mechanism might be promising in utilizing alternative therapeutic strategies that are based on TGF-β blocking.

Plasmacytoid dendritic cells (pDCs) are innate immune cells that secrete large amounts of interferon (IFN) and mediate Toll-like receptor (TLR)-induced inflammation in auto-immunity. pDCs have been recently detected in the sclerotic skin of patients with SSc. These cells were found to be chronically activated and characterized by an elevated expression of chemokine (C-X-C motif) ligand 4 (CXCL4) and IFN-α in a TLR8-dependent manner [28,50]. CXCL4 has been identified as a biomarker that is associated with severe disease and lung fibrosis in SSc [51]. pDCs have also been detected in bronchoalveolar lavage fluids of patients with SSc, and their levels were correlated with increased lung fibrosis [52]. Compared to patients with idiopathic pulmonary fibrosis (IPF), SSc-ILD-infiltrating pDCs exhibited a stronger IFN and stress response gene signature, suggesting that these cells demonstrate disease-specific mechanisms in SSc [53]. Recent research has provided mounting evidence suggesting that SSc is an IFN-driven disease [54]. Indeed, an elevated expression of type 1 IFN signaling (IFN-α, IRF5, IRF7, IRF8) and its inducible genes (IL-6, STAT1, STAT3) has been illustrated in tissue biopsies, the peripheral blood, and serum of SSc patients and was correlated with disease severity [55]. In the blood circulation, on the other hand, numbers of pDCs were decreased in SSc patients compared to healthy controls, probably due to their accumulation in the fibrotic skin [56]. Additionally, pDCs are well known for their prevalent antigen-presenting role. As we discussed, SSc is characterized by auto-immunity, and the presence of multiple anti-nuclear antibodies has been reported in patients’ serum. DNA topoisomerase I (topoI) is the most prevalent autoantibody in SSc and is correlated with increased disease severity and mortality [57]. Mice administered with topoI-loaded pDCs exhibited robust autoantibody production accompanied by long-term lung and skin fibrosis [58]. Furthermore, pDC depletion in the bleomycin mouse model attenuates skin and lung fibrosis and improves clinical scores. Interestingly, numbers of B and T lymphocytes were also reduced in mouse lungs, demonstrating an important role of pDCs in ongoing innate and adaptive immune abnormalities [50,52].

### 2.2. The Role of Adaptive Immunity in SSc

Activation of the innate immune system in SSc is essential in activating the adaptive immune response by presentation of antigens with parallel expression of danger-indicating molecules. This activation is mediated either by cell–cell interactions, or by the release of soluble mediators [59]. We will now briefly discuss the role of T and B lymphocytes in SSc pathogenesis (Table 1), in order to better explain the possible treatment options.

#### 2.2.1. T Lymphocytes

**Figure 3:**
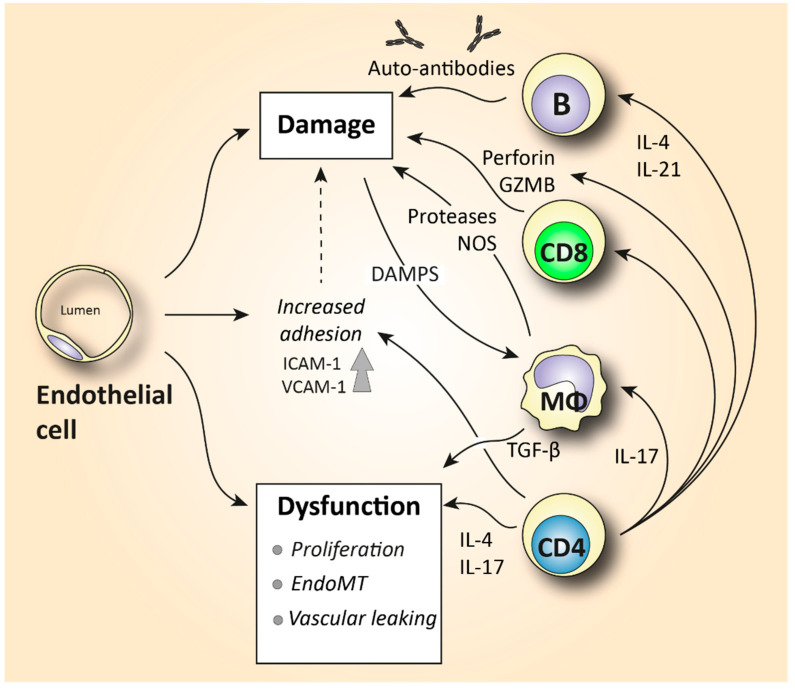
A simplified schematic illustration of the pathogenic mechanisms by which T cells, B cells, and macrophages may be involved in the vascular abnormalities that characterize SSc immunopathology. ICAM-1 = intercellular adhesion molecule 1; VCAM-1 = vascular cell adhesion molecule 1; EndoMT = endothelial-to-mesenchymal transition; GZMB = granzyme B; NOS = nitric oxide synthase; DAMPs = damage-associated molecular patterns; MΦ = macrophages; B = B lymphocyte; CD4 = T helper cell; CD8 = cytotoxic T cell; TGF-β = transforming growth factor-β.

The role of T helper lymphocytes is well characterized in SSc pathology. Th1, Th2, and Th17 subtypes have been paid the most attention. However, it should be noted that these subtype classifications have been derived from in vitro studies. In vivo, these subsets have been implicated in various types of pathogens. In chronic inflammatory diseases, they have been shown to exhibit a certain extent of plasticity and multifunctionality. In Th2-associated pathologies (including SSc), additional T cell subtypes such as Th9 and Th22 cells have also been characterized. However, the specific importance of them still remains to be determined. Studies implicating T helper cell subsets in SSc pathogenesis have mostly utilized older conventional research techniques such as immunohistochemistry in SSc skin and flow cytometry in patients’ blood. Elevated production of the Th2-associated cytokines IL-4, IL-13, and IL-10 and the Th17 signature cytokine IL-17 have been demonstrated in SSc tissues and the blood circulation [4,60,61,62,63] (Figure 2). These studies suggested that SSc is driven by Th2- and Th17-type mechanisms. Elevated IL-4 levels have been associated with increased collagen production in fibroblasts and higher production of TGF-β. TGF-β directly triggers collagen production and ECM deposition but also inhibits the expression of matrix metalloproteinases [64]. An increased level of IL-17 is implicated in fibrosis and SSc-like manifestations [65]. IL-17 not only increases the proliferation of fibroblasts but also promotes the expression of TNF-α and IL-1 from macrophages which, in turn, triggers collagen, IL-6, and PDGF production from fibroblasts. In addition, IL-17 triggers the endothelial cell production of IL-1, IL-6, intracellular adhesion molecule 1 (ICAM-1), and vascular cell adhesion molecule 1 (VCAM-1) [66] (Figure 3). These adhesion molecules further interact with circulating leukocytes and facilitate their migration to and accumulation at the fibrotic sites [67]. In addition to elevated Th2 and Th17 cytokines, SSc patients exhibit a reduced expression of the anti-fibrotic Th1 cytokine IFN-γ, and this further suggests a decreased anti-fibrotic capacity [63]. A T helper cell subset which could play a protective role in SSc pathology is regulatory T cells (Tregs). Tregs maintain immunological self-tolerance, and their depletion has been associated with spontaneous auto-immunity. A plethora of animal studies and a few clinical studies have shown a potentially beneficial effect of Treg administration in various auto-immune diseases [68]. In SSc, the majority of the published literature illustrates a decreased frequency and functional ability of circulating Tregs. However, data on the frequency of tissue-resident Tregs are scarce and contradictory [69]. Overall, the mechanisms of circulating and tissue-resident T helper cell subsets that drive fibrosis are not precisely determined. It is to be proven if mechanisms such as T cell tolerance, anergy, and exhaustion are protective or deleterious in fibrogenesis.

Novel techniques such as sc-RNAseq and confocal immunofluorescence microscopy have provided us with essential tools to gain a deep understanding of the phenotype and function of tissue-infiltrating T cells. Utilization of these newly available techniques reveals the heterogeneity of T cell responses and opens avenues for detecting patient-specific T cell subsets. For example, quantitative analyses of skin-resident cells showed that early diffuse cutaneous systemic sclerosis (dcSSc) skin was predominantly infiltrated by granzyme A-producing CD8+ and CD4+ CTLs. Th1, Th2, and Th17 cells were also detected but in much lower amounts. This study unveiled that CD4 T cells in SSc may directly induce cell death, a function that deviates from their conventional role in promoting effector immune responses from other lymphocytes. CD4+ and CD8+ CTLs exhibited the ability to drive fibrosis and contribute to vasculopathy (Figure 3) via the secretion of pro-inflammatory cytokines such as IL-1β and/or by inducing cytotoxicity-mediated apoptosis of stromal cells, leading to exuberant tissue remodeling [6]. The importance of CTLs in SSc pathogenesis is further supported by another recent study identifying the genome-wide expression of cytotoxic genes in SSc skin such as perforin and granzymes B, K, and H. Of note, the observed cytotoxic gene signature was positively correlated with skin thickness [70,71]. Furthermore, a prominent infiltration of IL-13-producing CD8+ CTLs was observed in skin biopsies early in SSc pathogenesis (<3 years), implicating a potentially important role of CTLs in the disease onset [72] (Figure 2). This notion is further supported by earlier studies that paid attention to the role of the CTL-mediated apoptosis induced by granzyme B in the initiation of systemic auto-immunity. The unique fragments generated by granzyme B degranulation represent an exclusive source of auto-antigens, and these self-protein fragments are recognized by autoantibodies in a subset of SSc patients [73].

Another example of a recently identified CD4+ T cell subset in SSc pathology is follicular helper T (Tfh) cells. Tfh cells are specialized in providing help to B cells in lymph nodes by stimulating proliferation, class switching, and somatic hypermutation. These cells have not only been found in lymph nodes but have also recently been detected in the blood circulation where they are referred to as T peripheral helper (Tph) cells [74]. A key cytokine in Tph function is IL-21. Strikingly, in SSc patients, elevated counts of Tph cells have been shown to promote plasmablast differentiation through an IL-21-mediated pathway [75]. Furthermore, increased infiltration of inducible T cell co-stimulator (ICOS)+ Tfh-like cells has been observed in the skin of SSc patients. The presence of these cells in the skin of sclerodermous graft-versus-host disease mice was strongly linked to increased skin fibrosis. Interestingly, both the depletion of ICOS+ (including Tph depletion) cells and neutralization of IL-21 exhibited a significant reduction in skin fibrosis. Tfh cells activate fibroblasts in vitro. Co-culture of Tfh and fibroblasts resulted in increased expression of α-smooth muscle actin (α-SMA) on the activated myofibroblasts [76]. α-SMA is a key marker of activated myofibroblasts, and its expression is linked to a fibrotic phenotype. The importance of skin-resident Tfh-like cells in the pathology of SSc is also supported by an sc-RNAseq study that unraveled the heterogeneity of T cell responses in patients’ skin biopsies. In this study, a distinct CXCL13+ Tfh-like subset that secretes factors promoting B cell responses and autoantibody production was only found in SSc-inflamed tissue [77]. In light of these new findings, developing drug strategies to target the newly described pathogenic T cell subsets is expected to set the milestones for potential personalized therapy in SSc.

#### 2.2.2. B Lymphocytes

B lymphocytes are the second major component of our adaptive immune response. Mounting evidence suggests that B cell homeostasis in the blood of SSc patients is aberrant. Autoantibody production caused by loss of self-tolerance is an important hallmark in SSc pathogenesis. Autoantibodies such as anti-DNA topoisomerase I, anti-centromere, and anti-RNA polymerase antibodies have been detected in the sera of more than 95% of scleroderma patients [78].

The prominent infiltration of B cells in SSc tissues including the skin and lungs suggests their involvement in the disease pathogenesis [79]. Similar to T cells, earlier studies revealed their presence in sclerotic skin by utilizing mainly immunohistochemistry techniques. In a recent sc-RNAseq study, a prominent B cell gene signature was detected in the skin of 69% of early dSSc patients [80]. In a different study, B cell infiltration was associated with skin progression in early diffuse disease. In this study, infiltration of plasma cells was also evident in the sclerotic skin [81]. Thus, B cell skin infiltration seems to be linked to skin progression early in the disease onset and in patients with dcSSc. However, the robustness of this correlation needs to be validated in a larger cohort of patients.

More specifically, B cells in SSc are characterized by elevated numbers of IL-6-producing effector B cells (Beffs) and decreased numbers of IL-10-producing regulatory B cells (Bregs). Because Bregs suppress and Beffs enhance the immune response through the production of cytokines, this change might impact the inflammatory process. Of note, in SSc, elevated IL-6 and reduced IL-10 levels have been detected in serum/plasma, which is possibly explained by this B cell imbalance. A possible cause for this change might be the altered presence of the cytokines B cell activating factor (BAFF) and a proliferation-inducing ligand (APRIL) in SSc blood [82]. BAFF and APRIL are potent activators of B cells. These cytokines stimulate the production of effector B cells and suppress the generation of regulatory B cells. Elevated serum levels of BAFF and APRIL have been documented in SSc and are correlated with skin thickening, disease severity, and increased IL-6 production by Beffs [83] (Figure 2). Interestingly, it has been demonstrated that BAFF inhibition decreased skin fibrosis in a murine model of SSc [84].

Importantly, not only the numbers of Beffs but also the phenotype of these cells is altered in SSc. To elaborate, SSc patients are characterized by an increased naïve (CD19+CD27-) and a reduced activated memory B (CD19+CD27+) cell phenotype that is accompanied by overexpressed pro-apoptotic and activation markers such as CD95, CD86, and human leukocyte antigen-DR isotype (HLA-DR) [4,85,86,87,88]. In addition, the cell surface phenotype of B cells is also changed in SSc. Significant overexpression of CD19 on the surface of B cells has been illustrated in SSc patients and was correlated with SSc-ILD [89,90]. However, in a recent study exploring lymphocyte subset aberrations between early dcSSc patients and healthy donors, the frequency of CD19+ B cells in the peripheral blood was similar [4]. CD19 is a positive B cell regulator that activates B cells through their B cell receptor (BCR) signaling. B cell hyperactivation through CD19-BCR signaling may be associated with the prevalent autoantibody production in SSc. Data from transgenic mouse models show that CD19-deficient mice have decreased serum autoantibodies, while overexpression of CD19 causes increased serum autoantibody production [91,92]. In addition, humans with homozygous mutations in the CD19 gene suffer from an antibody deficiency syndrome that impairs the response of mature B cells in antigen stimulation [93]. In conclusion, in addition to B cells which have been documented to stimulate fibrosis with the release of cytokines, intriguingly, autoantibodies with direct pro-fibrotic effects and autoantibodies against the PDGF receptor have also been documented in SSc [94]. These autoantibodies seem to induce fibrosis by facilitating the conversion of the fibroblast to the myofibroblast phenotype [95] (Figure 2).

**Table 1:** Function of the primary cell types implicated in SSc pathogenesis both in the blood circulation and at the site of fibrosis.

Cell Type	Function
Endothelial cell	Platelet adhesion activates fibrotic pathways. Increased microvascular permeability causes leukocyte adhesion to the endothelium, leading to increased inflammation [7].
Monocyte/Macrophage	A prominent M2 macrophage signature increases levels of pro-fibrotic cytokines such as IL-4, IL-6, and IL-13 and correlates with elevated tissue fibrosis [17,18,19,20].
Eosinophil	Elevated eosinophil counts in the peripheral blood are associated with severe lung disease and presence of skin ulcers [31,32,33].
Mast cell	Release of cytokines and growth factors such as IL-4, IL-6, IL-13, TNF-a, PDGF, and TGF-β activates myofibroblasts to produce collagen [37]. Tryptase and histamine release triggers fibroblast proliferation [38,39].
Innate lymphoid cell	Increased production of the ILC2 cytokines IL-25, IL-33, and TSLP in serum and skin mediates fibrosis in a TGF-β-dependent manner [43,44,45,46,47,48,49].
Plasmacytoid dendritic cell	Elevated numbers of CXCL4+- and IFN-a-producing pDCs in skin and lungs are involved in increased fibrotic manifestations mediated by TLR8 activation [28,50,52].
T lymphocyte	Skin is predominantly infiltrated by CD4+ and CD8+ cytotoxic T cells that produce pro-fibrotic cytokines and cause apoptosis to epithelial cells [6,72]. Increased IL-21-producing Tph cells promote plasmablast differentiation and increase activation of myofibroblasts [75,77]. In the peripheral blood, SSc patients are characterized by increased Th2 and Th17 numbers compared to healthy donors [4,60,63].
B lymphocyte	Elevated BAFF and APRIL are correlated with skin thickening [81,83]. Increased IL-6-producing Beffs increase inflammation, while decreased IL-10-producing Bregs exhibit a reduced capacity for immunosuppression [82]. In SSc peripheral blood, an increase in naïve and a decrease in activated memory B cells is observed compared to healthy controls [4,85,87,91].

## 3. SSc First-Line Anti-Inflammatory Treatment

Now that we have discussed the role of immune cells in SSc, we will address the efficacy of treatment approaches. According to current recommendations from the European League against Rheumatism (EULAR), methotrexate (MTX) is considered as a first-line treatment in early dcSSc [96]. Other therapeutic strategies include administration of synthetic corticosteroids or low-dose (2 mg/kg) administration of cyclophosphamide (CYC) for 1 year, continued by conservation therapy with mycophenolate mofetil (MMF) [97], or first-line therapy with MMF. These therapies represent the broad-spectrum anti-inflammatory drugs (Table 2) that have been extensively used in SSc, and thus we will start discussing them first.

### 3.1. Synthetic Corticosteroids

To begin, synthetic corticosteroids (CS) such as prednisolone, hydrocortisone, methylprednisolone, and dexamethasone are the oldest anti-inflammatory and immunosuppressive agents that have been extensively used in scleroderma and other rheumatic diseases. These compounds modulate the activation of all immune cells including macrophages and T and B lymphocytes but also affect the function of fibroblasts and endothelial cells. They still remain a cornerstone in the treatment of SSc as, due to their strong immunosuppressive and anti-inflammatory properties, they are able to halt inflammatory, vascular, and fibrotic manifestations (all key hallmarks of SSc pathogenesis) [98]. Retrospective case–control studies have shown that CS may improve muscle and joint inflammation and severe skin fibrosis [99,100,101], but to our knowledge, there is still no well-controlled, double-blind, placebo-controlled study to justify these indications. On the other hand, a high dose of prednisolone has been associated with an increased risk of scleroderma renal crisis [102,103,104]. This is a very severe and life-threatening complication and thus, in SSc, doses of prednisolone >10–15 mg are avoided. Administration of corticosteroids as monotherapy is not recommended. The addition of steroid-sparing agents such as MMF in the treatment scheme reduces doses of steroids along with their side effects and steroid requirements. In clinical practice, the use of CS in SSc is limited and restricted to the more inflammatory than pro-fibrotic manifestations such as arthritis and myositis. It is worth mentioning that CS still represent the mainstay of treatment for SSc patients with primary heart disease manifestations.

### 3.2. Methotrexate

MTX was developed as a cytotoxic folic acid analogue that inhibits purine and pyrimidine synthesis when administered at a high dosage. This means that it blocks cell proliferation, including that of immune cells. It was initially used to treat cancer and, at much lower dosages, rheumatoid arthritis (RA) [105]. Its extended use in RA suggests an important anti-inflammatory role. The exact mechanism of action at the dosage that is used for RA and SSc is not fully understood yet, but several mechanisms have been postulated to contribute to its anti-inflammatory function. It seems that MTX does not directly induce apoptosis of T cells and fibroblasts but rather increases their sensitivity to apoptosis by modulating cell survival signaling pathways. In contrast, MTX directly depletes monocytes in vitro by inducing their apoptosis [106]. Two small-scale randomized control trials (RCTs) in early dcSSC patients have evaluated the use of 15 mg MTX per week for 6 months [107,108]. The total skin score (TSS) was used as the primary endpoint to evaluate skin fibrosis. In both studies, a small but not statistically significant improvement in skin fibrosis was observed. Interestingly, continuation of the therapy up to 1 year showed a statistically significant improvement in the primary endpoints. Based on these observations, the EULAR [96] supported the use of MTX in skin manifestations in early dcSSC. However, a follow-up study [107] did not confirm the initial promising results in the long run. Taken together, data evaluating the effectiveness of MTX in reducing skin fibrosis are contradictory, and thus treatment guidelines supporting the use of MTX are mostly based on the positive experience of expert physicians with MTX.

### 3.3. Cyclophosphamide

CYC is another cytotoxic agent that targets fast-dividing cells such as tumor cells and proliferative T and B cells. Its use in auto-immune diseases is attributed to inhibition of regulatory and helper T cell proliferation, leading to their suppression. Suppression of helper and regulatory T cells leads to declined gene expression of pro-inflammatory cytokines, such as interleukin-2, and attenuated production of the fibrogenic TGF-β and the immunoregulatory IL-10, respectively [109,110]. In addition, lymphocyte depletion can decrease the production of antibodies (including autoantibodies) [111]. These observations suggest a potential role of CYC in diminishing fibrosis in patients with SSc-ILD. In the scleroderma lung study (SLC), 158 patients with SSc-ILD received 2 mg/kg of CYC or placebo orally for 12 months. The primary endpoint was the absolute difference in predicted forced vital capacity (FVC) which is a prediction of the patient’s lung function. Secondary endpoints included measurement of skin thickening with the modified Rodman skin thickness score (mRSS) and monitoring of lung function with high-resolution computed tomography (CT) scans. It was observed that the CYC group exhibited reduced skin fibrosis and thoracic fibrosis after the 12-month treatment compared to baseline (*p* = 0.014) [112,113]. All in all, CYC showed a measurable but small effect on improving the lung function of SSc patients. However, the use of CYC is limited due to severe toxicity notifications including leucopenia and thrombocytopenia. Thus, according to the EULAR [96], the usage of CYC is only recommended in patients with progressive ILD.

### 3.4. Mycophenolate Mofetil

Another broad-spectrum agent that has been used in SSc treatment is MMF. MMF causes selective depletion of guanosine nucleotides in T and B lymphocytes. It inhibits their proliferation and therefore suppresses immune-associated responses and antibody formation [114]. Indeed, SSc patients receiving MMF exhibit decreased numbers of T helper cells (implicated in various SSc pathogenic manifestations) compared to patients with no immunosuppressive treatment [115]. Strikingly, MMF treatment inhibits the infiltration of myeloid cells including tissue-resident macrophages in the skin of patients with SSc. Furthermore, treatment with MMF is strongly associated with a reduced inflammatory gene signature in the sclerotic skin [116]. Although MMF has been traditionally regarded as a lymphocyte-targeting drug, in vitro and clinical evidence shows its anti-fibrotic capacity by decreasing the proliferation of human fibroblasts [117]. The results from the Scleroderma Lung Study I and II suggest the efficacy of MMF in SSc patients with severe lung fibrosis. More specifically, treatment with 3 gr/day of MMF for 2 years resulted in a significantly improved percentage of predicted FVC and mRSS [118]. The effect of MMF has also been evaluated on patients with mild SSc-ILD (FVC ≥ 70% predicted). In this double-blind, placebo-controlled, randomized clinical trial [119], MMF was well tolerated while exhibiting an observed but not statistically significant improvement in FVC and mRSS scores. In the Scleroderma Lung Study, previous results of MMF were compared with results from oral CYC administration (2.0 mg/kg per day), followed by placebo administration for 1 more year. The adjusted percentage of predicted FVC after 24 months improved by 2.19 with MMF and 2.88 with CYC. However, no statistically significant differences among the two treatment groups were observed, and thus the superiority of MMF compared to CYC is not justified. In the European Scleroderma Observational Study (ESOS), MMF was further compared with MTX, CYC, or “no immunosuppressant” [1]. This was a multicenter, prospective, observational cohort of 326 patients with early dcSSc (up to 3 years of onset of skin thickening) with a 24-month duration. After 12 months of treatment, a statistically significant reduction in mRSS was observed in all groups compared to baseline measurements. To sum up, results from clinical trials regarding the anti-fibrotic efficacy of MMF are also conflicting, and there are no signs revealing its superiority to MTX and CYC. Similar to MTX, the recommendation for first-line MMF treatment in patients with SSc-associated ILD has been based on positive clinical experience with the drug, and its beneficial safety profile [120]. The use of MMF in improving lung fibrosis of SSc-ILD has also been supported by a large number of small retrospective cohort studies [121,122,123,124,125].

### 3.5. Autologous Hematopoietic Stem Cell Transplantation

ASCT has been utilized in treating auto-immune disorders resistant to conventional immunosuppressive therapy for more than 20 years now [126]. ASCT is the only potential disease-modifying treatment in SSc, and it has been proposed for patients with early and rapidly progressive dcSSc who have a high mortality prognosis, but in whom advanced organ involvement has not started yet [127]. Autologous stem cell transplantation begins with the isolation of the patient’s CD34+ cells. Then, B and T cells are depleted using a high dose of cyclophosphamide and anti-thymocyte globulin. The last step includes the transplantation of the patient’s stem cells that were isolated, resulting in the repopulation of the lymphocytes [128]. According to clinical data, ASCT shows promising results in improving mortality rates, reducing skin thickness, and enhancing lung function [129,130]. Two randomized clinical trials evaluating the safety and efficacy of ASCT in SSc have been completed thus far. First, in the Cyclophosphamide or Transplantation (SCOT) trial (NCT00860548), it was observed that myeloablative CD34+-selected ASCT was more effective in diminishing skin fibrosis than CYC administration alone. However, the mortality rates of the participants during the first year after the transplantation were as high as 10%. Treatment-related deaths as well as cancer and infection were the causes of death in these participants. Among them, infection was the leading cause, due to the suppression of the immune system. Furthermore, the possibility of relapsing was also prevalent. Secondly, in the Autologous Stem Cell Transplantation International Scleroderma (ASTIS) study [131], ASCT was compared with CYC pulse therapy in 156 rapidly progressive diffuse SSc patients. In the first year, eight patients from the ASCT group compared to none in the CYC group died. However, parameters such as long-term event-free survival, overall survival, mRSS score, and FVC were significantly improved in the ASCT-treated patients. The increased mortality rates counteract transplantation’s benefit, putting experts in a difficult judging position [132]. However, to date, no disease-modifying anti-rheumatic drug has been shown to effectively reduce patients’ morbidity in the long term. ASCT, on the other hand, has shown a remarkable reversal of skin fibrosis accompanied by improved lung and internal organ function. In view of the outcomes of the previously described RCTs, the new EULAR recommendations [96] suggest that experts consider ASCT for the treatment of rapidly progressive SSc patients at risk of organ failure. It is believed that treatment-related mortality can be reduced by a more careful exclusion of patients with compromised heart function and by applying ASCT in earlier stages of the disease, enabling selected patients to benefit from this treatment. There are currently four ongoing clinical trials evaluating the effectiveness and safety of ASCT in SSc (NCT01895244, NCT01413100, NCT04464434, NCT03630211). The results from these studies are expected to shed light on the safety and efficacy of autologous ASCT in a better stratified SSc patient population.

**Table 2:** Clinical trials conducted for the evaluation of broad-spectrum treatment in SSc.

Drug	Target	Type of Trial(s)	Duration (Months)	Patients	Results
Methotrexate (MTX)	Exact anti-inflammatory role is unknown	Multicenter, double-blind			
		1. RCT [108]	1. 6	1. 29 early dSSc	1. Mean TSS 21.61 at baseline, 19.96 (*p* = 0.135) 6 months after
		2. RCT [107]	2. 12	2. 71 early dSSc	2. Mean TSS 18.3 at baseline, 14.5 (*p* = 0.027) 12 months post-treatment
Cyclophosphamide (CYC)	Inhibition and suppression of T helper and regulatory T cells	Double-blind, RCT (SLC) [112]	12	158 SSc-ILD	2. 53% (*p* < 0.03) improvement in predicted FVC and 3.02 (*p* = 0.08) unit improvement in mRSS in CYC’s favor
Mycophenolate mofetil (MMF)	T and B cell depletion	1. Double-blind, RCT (SLC II) [118]	1. 24	1. 69 SSc-ILD	1. Percentage of predicted FVC improved from 67 to 75, and mRSS decreased from 14.5 at baseline to 10 24 months post-treatment
		2. Double-blind RCT [119]	2. 6	2. 41 mild SSc-ILD	2. No statistically significant improvement in mRSS and FVC scores
Autologous hematopoietic stem cell transplantation (ASCT)	Depletion of T and B cells, followed by stem cell transplantation	1. Open-label, multicenter RCT (SCOT) (NCT00860548)	1. 54	1. 75 severe SSc	1. ASCT more effective in diminishing skin fibrosis compared to CYC (−19.9 vs. −8.8, *p* < 0.001) and shows greater event-free survival
		2. Open-label, multicenter RCT (ASTIS) [131]	2. 24	2. 156 early dcSSc	2. Overall survival, mRSS, and FVC significantly improved with ASCT compared to CYC (67% of 1404 pairwise comparisons in favor of ASCT vs. 33% in CYC, *p* = 0.01)

## 4. Evaluation of Cell-Specific Anti-Inflammatory Treatment

On the road towards personalized therapy in SSc, specific elimination of pathogenic innate or adaptive immune cell populations could be promising in alleviating fibrosis and reducing the morbidity of the side effects of broad immunosuppression. Of note, there are no clinical trials evaluating drugs depleting innate immune cell populations. On the other hand, several trials (Table 3) have evaluated the efficacy of B and T cell-depletive therapies in SSc, and thus we begin with addressing treatments targeting these cells first [133].

### 4.1. B Cell-Specific Treatment

To begin with B cell-specific treatment, rituximab (RTX), belimumab, and inebilizumab are the three representative monoclonal antibodies that have been used.

#### 4.1.1. Rituximab

**Figure 4:**
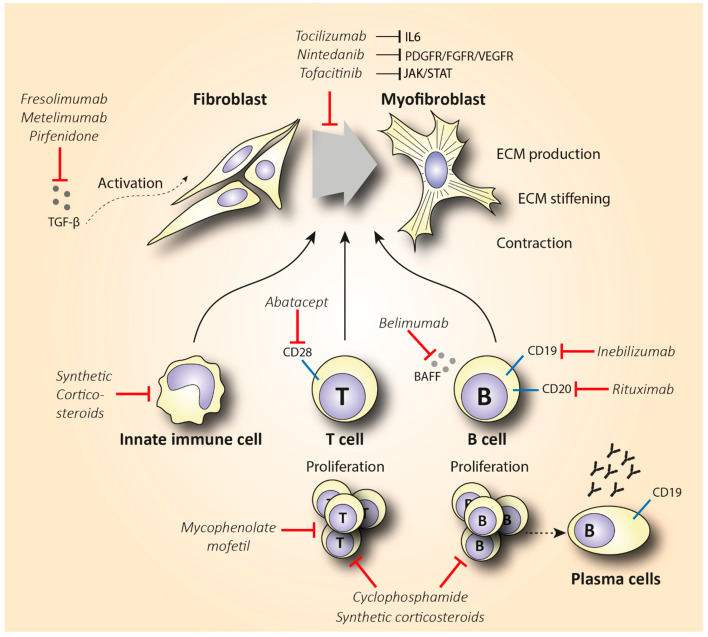
Schematic representation of the mechanism of action of synthetic corticosteroids (CS), mycophenolate mofetil, cyclophosphamide, inebilizumab, rituximab, belimumab, abatacept, nintedanib, tocilizumab, tofacitinib, fresolimumab, metelimumab, and pirfenidone in reducing fibrosis in SSc. Abbreviations: ECM = extracellular matrix; TGF-β = transforming growth factor-β.

Rituximab is a chimeric anti-CD20 monoclonal antibody that binds to both immature and mature B lymphocytes expressing CD20 on their surface (including the pathogenic Beffs) and eliminates them (Figure 4). Of note, RTX does not consistently succeed in depleting B cells in tissues [134,135], and antibody-producing plasma cells are not targeted by RTX, since they lack the CD20 surface antigen [136]. Indeed, in a small, randomized, double-bind, placebo-controlled trial, treatment with RTX was not associated with reduced fibrosis but caused significant depletion of naïve and memory B cells in the peripheral blood and scleroderma-associated dermal lesions [137]. As expected from RTX’s mode of action, various plasma cells and autoantibody titers did not show a statistically significant change after treatment. The potential anti-fibrotic effect of RTX is supported by histopathological observations suggesting a reduced dermal hyalinized collagen content and myofibroblast count [138]. As we discussed, IL-6 is a cytokine largely produced by activated B cells that shows pro-fibrotic capacity. In a small, open-label study where RTX significantly improved patients’ skin scores, it was observed that patients had elevated basal levels of IL-6 in biopsies from their skin lesions. IL-6 levels decreased from 3.7 ± 5.3 pg/mL at baseline to 0.6 ± 0.9 pg/mL (*p* = 0.02) six months post-treatment. Furthermore, after treatment 7/9 patients exhibited a complete depletion of B cells in skin lesions. Thus, interestingly, the RTX-associated skin thickness was correlated with B cell depletion and IL-6 reduction in patients’ skin [139]. This observation further empowers the mechanism and role of RTX in skin fibrosis.

The potential efficacy of treating SSc patients with RTX has been extensively evaluated by Daoussis and colleagues. In an open-label, randomized, 1-year study, weekly administration of RTX (375 mg/m^2^) for 4 weeks at baseline and after 6 months on top of each patient’s standard treatment compared to standard treatment alone was assessed. Patients that received RTX showed a significantly increased median percentage of FVC accompanied by a further improvement in the diffusing capacity for carbon monoxide (DLCO). Furthermore, skin improvement was also evident when evaluated both clinically and histologically [140]. In a more recent (2017) multicenter, open-label study, the same group of researchers compared administration of 4 infusions of RTX at a dose of 375 mg/m^2^ once weekly every 6 months with controls receiving traditional therapies (MTX, azathioprine, MMF). In a 4-year follow-up, the RTX group showed a reduced mRSS score of 14.72 ± 10.52 compared to 17.78 ± 9.48 in the control group (*p* = 0.31). Patients enrolled in this study exhibited a significant improvement in FVC and stabilization of pulmonary function tests (PFTs) [141]. In the latest open-label, randomized, head-to-head clinical trial, 60 anti-scl70-positive early dcSSc patients randomly received either monthly pulses of CYC (500 mg/m^2^) or 1 g of RTX at 0 and 15 days. In the RTX group, there was a significant improvement in the percentage of FVC, while patients receiving CYC experienced deterioration of their lung function. Both groups exhibited a similar improvement in skin scores, but the safety profile of RTX was better [142].

Furthermore, B cell depletion may be an efficient and well-tolerated adjuvant treatment for SSc-associated pulmonary arterial hypertension (SSc-PAH). In a multicenter, double-blind, randomized, placebo-controlled, proof-of-concept trial, 57 SSc patients on standard medical therapy received two doses of either 1 gr RTX or placebo in a 2-week interval [143]. To evaluate the effect on PAH, the change in the 6 min walk distance (6MWD) was the primary outcome. Twenty-four weeks after treatment, patients in the RTX arm exhibited an estimated change of 23.6 ± 8.8 m compared to 0.4 ± 9.7 m in the control arm (*p* = 0.03). The beneficial results of RTX in diminishing skin fibrosis have also been demonstrated in a number of case or pilot studies [144,145,146,147,148]. Most of these studies, however, were open label, often lacked a control group, and included heterogeneous patient populations and concurrent treatments. Interestingly, the fact that RTX shows positive results in reducing skin fibrosis and alleviating PAH and ILD, together with being more potent than traditional medications and well tolerated in all studies, suggests a promising role in scleroderma medication options. However, a phase III randomized control study will be required to verify its safety and efficacy in patients.

#### 4.1.2. Inebilizumab and Belimumab

Since depletion of CD20+ B cells does not consistently deplete B cells in tissues and does not eliminate plasma cells, developing monoclonal antibodies that target the bone marrow-resident pro- and pre-B cell co-receptor CD19 might be a more effective B cell-depletive approach (Figure 4). The efficacy of a humanized anti-CD19 monoclonal antibody, inebilizumab (MEDI-551), in reducing SSc fibrosis was assessed in a phase I randomized, double-blind, placebo-controlled study [149]. This therapy effectively depleted both B cells and plasma cells in the skin and blood of SSc patients in a dose-dependent manner. Furthermore, an improved skin score was observed in all patients, suggesting a role of inebilizumab in halting skin fibrosis. Strikingly, patients with an elevated plasma cell signature at inclusion showed greater improvement in mRSS compared to the patients that had normal or lower plasma cell counts [150].

Belimumab is another human monoclonal antibody targeting the soluble BAFF cytokine, a member of the tumor necrosis factor ligand superfamily that was a breakthrough in the treatment of systemic lupus erythematosus (Figure 4). This monoclonal antibody prevents the binding of BAFF to receptors on B cells and inhibits the survival of B cells, including the autoreactive ones. It further decreases the differentiation of B cells into immunoglobulin-producing plasma cells [151]. In a randomized, double-blind, placebo-controlled, pilot trial, 20 patients with dcSSc recently treated with MMF (1 gr twice a day) were administered 10 mg/kg of belimumab i.v., or placebo. Belimumab administration improved the mRSS score from 27 to 18 (*p* = 0.039), while the improvement in the placebo group was from 28 to 21 (*p* = 0.023). Patients with improved mRSS exhibited a significant reduction in both B cell and pro-fibrotic gene expression. However, the results of this small pilot study were not statistically significant, and larger trials should be conducted in order to make treatment recommendations [152]. A study evaluating the combination therapy of belimumab and RTX in SSc is currently pending (NCT03844061).

### 4.2. T Cell-Specific Treatment

The elevated infiltration of cytotoxic T cells in SSc-affected skin and lungs, along with the increased Th2 pro-fibrotic cytokine levels in the serum of SSc patients, suggests that targeting T cells could be a promising treatment option in SSc. T cell-specific treatment has shown encouraging results in other inflammatory diseases such as RA [153]. The biologicals that have been used either directly affect T cell activity by reducing their number, or they indirectly interact with cytokines expressed by T cells. Abatacept is the main representative of the former category, and it causes T cell deactivation by blocking CD28 (Figure 4), a molecule that is essential for T cell activation mediated by antigen-presenting cells (APCs) [154]. CD28 knockout mice exhibited decreased fibrosis in comparison with the wild type in bleomycin-induced fibrosis [155]. In 2015, the efficacy of abatacept in attenuating fibrosis was examined in a placebo-controlled RCT that included 10 patients with dcSSc [156]. Five out of seven patients that received abatacept showed a ≥30% decrease in mRSS, while this improvement was shown in one out of the three controls. Interestingly, patients with decreased mRSS also showed reduced CD28 gene expression along with decreased expression of genes associated with T cell proliferation. This empowers the proposed mechanism of abatacept’s action. Recently, a phase II, multicenter, double-blind, randomized, placebo-controlled trial assessed the safety and efficacy of subcutaneous administration of abatacept in 88 dcSSc patients [157]. The results failed to show a statistically significant improvement in mRSS (primary outcome) in the abatacept arm, but secondary outcomes reflecting patients’ disability and quality of life were significantly improved in favor of the abatacept group. Of note, patients were stratified into inflammatory, normal-like, and fibroproliferative subgroups based on molecular gene expression data of their skin biopsies. Patients with an inflammatory intrinsic gene expression showed a larger decrease in mRSS compared to the other groups. Interestingly, the improvement in the skin score was associated with decreased gene expression of immune pathways including cytotoxic T lymphocyte-associated protein 4 (CTLA-4) and CD28 (targets of abatacept). These results are novel regarding the potentially targeted mechanism of action of abatacept as an inflammation immunomodulator. Furthermore, abatacept exhibits a good safety profile and seems to decrease joint involvement and related disability [158,159]. Based on these studies, abatacept seems to be effective in treating scleroderma-associated joint inflammation, but studies with a larger number of participants (phase III) are required to evaluate its anti-fibrotic effect.

**Table 3:** Representation of the studies evaluating the role of B and T cell-specific treatments in SSc.

Drug	Target	Type of Trial(s)	Duration (Months)	Patients	Results
Rituximab (RTX)	Anti-CD20 B cell depletion	1. Double-blind, RCT [137]	1. 24	1. 16 early SSc	1. B cell depletion in blood did not improve mRSS and FVC scores
		2. Open-label trial [139]	2. 54	2. 9 dcSSc	2. Median decrease in mRSS of 43.3% (*p* = 0.001) accompanied by reduction in IL-6 levels
		3. Open-label RCT [140]	3. 24	3. 14 SSc	3. FVC improved by 10.25% in RTX and reduced by 5.04% in the placebo group (*p* = 0.0018). RTX arm: mRSS improvement of 13.5 vs. 8.37 in placebo, 12 months post-treatment (*p* < 0.001)
		4. Multicenter, open-label trial [141]	4. 48	4. 51 SSc-ILD	4. No significant change in mRSS, but lung function improved significantly
		5. Open-label RCT [142]	5. 6	5. 60 early dSSc	5. Significant improvement in RTX vs. CYC in median percentage of FVC (67.52 vs. 58.06, *p* = 0.003), but not in mRSS scores
		6. Multicenter, double-blind RCT [143]	6. 6	6. 57 SSc-PAH	6. RTX arm: the improvement in median 6MWD was 25.5 m compared to 0.4 m in placebo (*p* = 0.03) after 48 weeks
Inebilizumab (MEDI-551)	Anti-CD19 B cell depletion	Multicenter, double-blind RCT [149]	3	28 SSc	Depletion of B and plasma cells was correlated with improved mRSS and reduced expression of fibrotic genes in skin biopsies
Belimumab	Inhibition of B cell survival by blocking BAFF	Double-blind RCT [152]	13	20 dcSSc	mRSS score improved from 27 to 18 (*p* = 0.039), while in placebo group from 28 to 21 (*p* = 0.023)
Abatacept	CD28 blocking T cell depletion	1. RCT [156]	1. 10	1. 6 dcSSc	1. 5/7 patients and 1/3 controls showed >30% improved mRSS
		2.Multicenter, double-blind RCT [157]	2. 12	2. 88 dcSSc	2. No significant improvement in mRSS, but secondary outcomes related to quality of life improved significantly

## 5. Targeting Cytokine Production

An alternative option to T cell treatment may be targeting cytokines and growth factors (Table 4), such as IL-6, IL-1, IL-2, and TGF-β, that are produced by multiple pathological cell types in SSc. Tocilizumab (TCZ) is a monoclonal antibody that inhibits IL-6 signaling, by blocking the IL-6 receptor (Figure 4), and is effective in treating patients with juvenile idiopathic arthritis and RA [160,161]. Multiple studies have evaluated the use of TCZ in systemic sclerosis as well. Among these studies, there is one open-label, phase II, randomized controlled study (faSScinate) [162] that evaluated the efficacy and safety of a 24-week treatment with TCZ in reducing fibrosis. The observed difference of 2.70 units in mRSS was not statistically significant (*p* = 0.0579). However, the interesting findings of this study arise from a probable correlation between decreased IL-6 levels and TGF-β function. More specifically, researchers cultured fibroblasts from patients’ skin biopsies at baseline and 24 weeks after treatment and showed that the IL-6 reduction was correlated with decreased TGF-β-related gene expression, suggesting a role of TCZ in reducing fibrosis [163]. The European Scleroderma Trials and Research (EUSTAR) observational study also evaluated the use of TCZ in SSc-associated joint and muscle inflammation, compared to abatacept. Similar to abatacept, the reduction of 3 units in mRSS between baseline and post-treatment was not significant (*p* = 0.109), but a significant improvement in joint function was observed [158]. A number of case series have shown similar results [164,165,166,167,168]. Post hoc analysis of the results from the faSScinate study [162] showed that considerably fewer patients treated with TCZ compared to placebo exhibited a decline in their lung function. These participants were also characterized by a reduced expression of pro-fibrotic genes, suggesting that TCZ treatment may be associated with preserved lung function. These promising results, together with the unmet medical need for effective fibrosis-reducing treatment, supported the exploration of TCZ in a phase III clinical trial (focuSSed) [169]. In this very recent randomized, double-blind, placebo-controlled, multicenter trial, 210 dcSSc patients were administered subcutaneous TCZ (162 mg) or placebo weekly for 48 weeks. The reduction in mRSS at the endpoint compared to baseline levels was −6.14 for TCZ and −4.41 for placebo, with the adjusted difference of −1.73 not being able to show statistically significant results (*p* = 0.10). Interestingly, the change in the FVC percentage predicted at week 48 was in favor of the TCZ arm, expressed as a difference in the least squares mean of 4.2 (*p* = 0.0002). These results further support the protective role of TCZ in preserving lung function. This was later investigated by stratifying the same patients according to the level of lung involvement [170]. Lung involvement was evaluated by serial spirometry, high-resolution chest CT, and quantitative interstitial lung disease (QILD) and fibrosis scores. Strikingly, TCZ was effective in stabilizing and thus preventing the progression of early SSc-ILD in all patient groups, irrespective of the severity of lung involvement. These results likely highlight the importance of targeting the immunoinflammatory, early fibrotic stage of SSc pathology. Taking everything together, TCZ does not seem to significantly improve skin fibrosis, but it seems to serve as a safe treatment option to preserve patients’ lung function. Thus, TCZ has now been approved by the US Food and Drug Administration (FDA) to treat SSc-ILD.

Based on the elevated levels of IL-1 and IL-2 and their role in inflammation and fibrosis in SSc, the potential effect of the monoclonal antibodies rilonacept and basiliximab in tackling SSc-associated fibrosis has been examined lately. Rilonacept is an anti-inflammatory treatment that has been approved by the FDA as a therapy for cryopyrin-associated periodic syndromes [171]. Its potential use in SSc depends on the fact that it blocks IL-1β signaling by binding with IL-1β and preventing its reaction with cell surface receptors, therefore reducing IL-1-triggered inflammation. The results from a double-blind, placebo-controlled, randomized trial did not support this hypothesis, as no changes were observed in mRSS between treatment and placebo. In addition, the researchers measured IL-1-regulated gene expression from explants obtained from patients’ skin, and similarly, no difference was found [172]. Regarding IL-2 expression, basiliximab is an anti-CD25 monoclonal antibody that inhibits the IL-2 receptor and is used for the treatment of kidney allograft rejection as it inhibits the activation and proliferation of T cells [173]. The selective inhibition of IL-2 cytokines seems promising in counteracting the Th2 predominance in SSc lesions, but is this associated with reduced fibrosis? The results from a case study in which a patient with early dcSSC was treated with a combination of CYC, prednisolone, and basiliximab showed a decrease in mRSS from 24 at baseline to 19 six months post-treatment [174]. The same research group, 4 years later, conducted a small-scale open-label study including 10 patients with dcSSC [175]. The median mRSS was reduced from 26/51 to 11/51 at week 68 (*p* = 0.015). Furthermore, the median predicted FVC between baseline and 44 weeks after treatment increased from 82.1% to 88.4%. The observed trend towards an improvement in skin disease cannot be attributed to basiliximab alone as the patients also received other immunosuppressive treatments and there was no control group to compare with. Therefore, whether basiliximab can attenuate fibrosis is still unclear.

Another important inflammatory and pro-fibrotic mediator in SSc is TGF-β. Currently, drugs targeting the TGF-β pathway are under investigation mainly for diseases such as cancer. In SSc, the possible efficacy in reducing fibrosis by blocking TGF-β has been examined with the use of the monoclonal antibodies fresolimumab and metelimumab (Figure 4). In a small-scale, open-label, single-center study, fresolimumab, a high-affinity TGF-β-inactivating monoclonal antibody, reduced TGF-β-dependent gene expression in skin biopsies. Furthermore, fresolimumab administration was accompanied by an average reduction of 8 units in mRSS at 11 weeks post-treatment [176]. On the other hand, metelimumab’s administration did not show similar encouraging effects. The effect of metelimumab has been examined in a randomized, double-blind, placebo-controlled trial in 45 patients with early (<18 months) dcSSc. All patients experienced an improvement in mRSS, but this improvement was not statistically significant (*p* = 0.49). Furthermore, the drug’s administration was related to an increased morbidity. A large number of severe adverse effects were reported, with skin ulceration and worsening of breathlessness being the most prominent [177]. With the provided results, TGF-β targeting exhibits conflicting results. Currently, several other compounds that target this pathway are under investigation. Those that target TGF-β signaling by inhibiting integrin expression have, thus far, shown promising results in reducing fibrosis in animal models [178].

Pirfenidone is a small molecule agent that interferes with TGF-β signaling and has potential interest in attenuating SSc-associated fibrosis. Pirfenidone is a pyridine derivative that is widely used in IPF, as it reduces fibroblast proliferation and TGF-β-induced collagen production in primary skin fibroblasts [179] (Figure 4). However, the molecular target of pirfenidone remains unknown. Of note, only one open-label, phase II study (LOTUSS) has evaluated the safety and tolerability of pirfenidone in patients with SSc-ILD. The results of this study did not confirm the IPF observed beneficial effect of pirfenidone in the treatment of ILD in scleroderma patients, as there was no difference in the predicted FVC between baseline and post-treatment [180]. This inability to improve clinical primary outcomes is also reflected molecularly, since serum levels of TNF-α and TGF-β did not significantly differ between treated and untreated patients. These outcomes are in line with the neutral effect on lung function reported by retrospective case studies of SSc patients with ILD that were treated with pirfenidone [181,182]. Given the documented efficacy of pirfenidone in IPF and the overlapping pathogenic manifestations between IPF and SSc-ILD, further investigation of the potential anti-fibrotic effect of pirfenidone in patients with SSc-ILD is needed. In this regard, two phase II and one phase III, multicenter, double-blind, randomized, placebo-controlled trials that are evaluating the efficacy of pirfenidone in diminishing skin fibrosis in SSc-ILD are currently recruiting and may reinforce the potential anti-fibrotic role of pirfenidone in the near future (NCT03068234, NCT03221257, NCT03856853).

**Table 4:** Clinical trials and other studies that evaluated the role of cytokine targeting treatment in SSc.

Drug	Target	Type of Trial(s)	Duration (Months)	Patients	Results
Tocilizumab (TCZ)	Inhibits IL-6 signaling	1. Open-label RCT (faSScinate) [162]	1. 24	1. 87 early SSc	1. Insignificant change in mRSS, but IL-6 reduction was correlated with decreased TGF-β expression
		2. EUSTAR observational study [158]	2. 5	2. 189 SSc-polyarthritis and myopathy	2. No statistically significant change in mRSS, but remarkable improvement in joint function
		3. Multicenter, double-blind RCT (focuSSed) [169]	3. 12	3. 210 dcSSc	3. Change of −1.73 in mRSS between treated and placebo groups was not statistically significant (*p* = 0.10), but the 4.2% improvement in predicted FVC was (*p* = 0.0002)
Rilonacept	Blocks IL-1b signaling	Double-blind RCT [172]	5 weeks	19 dcSSc	No changes in mRSS between treatment and placebo
Basiliximab	Anti-CD25-mediated inhibition of IL-2 inhibits T cell activation and proliferation	Small-scale, open-label study [175]	17	10 dcSSc	Median mRSS reduced from 26/51 to 11/51 at week 68 (*p* = 0.015) Median predicted FVC between baseline and 44 weeks after treatment increased from 82.1% to 88.4%
Fresolimumab	Blocks TGF-β signaling	Small-scale, open-label study [176]	6	15 dcSSc	Reduction of 8 units in mRSS score (*p* < 0.001) Reduced expression of TGF-β-regulated genes in skin biopsies (*p* < 0.049)
Metelimumab	Blocks TGF-β signaling	Double-blind RCT [177]	6	45 early SSc	No statistically significant change in mRSS scores
Pirfenidone	Reduces fibroblast proliferation and TGF-β-induced collagen production in primary skin fibroblasts	Open-label, phase II study (LOTUSS) [180]	4	63 SSc-ILD	No difference in the predicted FVC between baseline and post-treatment

## 6. Emerging Therapies with Tyrosine Kinase Inhibitors

Tyrosine kinases are enzymes that use adenosine triphosphate (ATP) to transfer a phosphate group to intracellular proteins. Phosphorylation of proteins by kinases is vital for signal transduction and regulation of cell proliferation, differentiation, migration, and development. The pathological activation of tyrosine kinases (TKs) is crucial in multiple disease processes such as carcinogenesis, vascular remodeling, and fibrogenesis [183,184]. Furthermore, in inflammatory lesions, tyrosine kinases are also activated through over-production of growth factors and/or cytokines from the tissues [185]. Several current studies suggest that inhibiting tyrosine kinases may lead to an effective anti-inflammatory therapy. Of note, targeting TK activity is feasible with monoclonal antibodies designed to target the extracellular domains of the receptors, or with small molecules that enter the cytoplasm and bind to intracellular catalytic domains of both receptor and non-receptor TKs [186] (Table 5).

### 6.1. Indolinone-Derived Small Molecule Tyrosine Kinase Inhibitors

Imatinib mesylate is the principal compound among the small molecule TK inhibitors and was approved by the FDA in 2001 for the treatment of chronic myelogenous leukemia, in order to block the Ab1 kinase activity [187]. Its use in SSc has been examined due to its ability to inhibit the PDGF receptor and TGF-β signaling pathways [188]. Several case studies [188,189,190,191,192] have examined the efficacy and tolerability of imatinib in scleroderma patients. Moderate improvement in skin scores and predicted FVC has been reported, along with a large number of adverse effects. From a mechanistic point of view, imatinib successfully depleted patients’ pDCs, immune cells that play an important role in the disease’s pathogenesis [192,193]. Of note, treatment with imatinib exhibited a reduced amount of pathogenic IL-4-producing T cells in bronchoalveolar lavage of SSc patients, which was accompanied by elevated numbers of total T helper cells. Based on this observation, it could be suggested that imatinib’s anti-fibrotic capacity could be mediated via shifting the Th2 response to a non-type 2 T helper phenotype [190]. In an open-label, multicenter study, 16 out of 27 patients with early dcSSc completed a six-month imatinib administration. The mean decrease in mRSS was 21% compared to baseline; however, five patients exhibited severe adverse effects including generalized edema, erosive gastritis, anemia, upper respiratory tract infection, neutropenia, and neutropenic infection [193]. Therefore, based on the moderate improvement and the severe adverse effects that were observed, the use of the imatinib’s analog nilotinib was proposed.

Nilotinib has the same mechanism of action as imatinib, but it is 20–30-fold more potent and has a more favorable toxicity profile [194]. The use of nilotinib in SSc has been tested in an open-label, single-arm, phase II trial that included 10 scleroderma patients. In this trial, nilotinib was well tolerated by the majority of the participants (7/10), with only a few cases of increased edema and mild QTc prolongation being reported. Furthermore, it was observed to be promising in reducing mRSS scores in patients with early and active disease (reduction of 16%, *p* = 0.02, and 23%, *p* = 0.01, at 6 and 12 months post-treatment, respectively) [195]. Furthermore, data from skin gene expression profiling showed that patients responding to treatment were characterized by elevated TGF-β signaling. Interestingly, the expression of TGF-β signaling genes was significantly reduced after these patients were treated with nilotinib. This observation empowers the mechanism of action of nilotinib and supports its potential anti-fibrotic effect. However, the progression into phase III trials will help to validate its efficacy in reducing fibrosis and also determine its limitations concerning potential side effects.

The last indolinone-derived small molecule inhibitor that will be discussed is nintedanib. Nintedanib inhibits a plethora of molecules implicated in fibroblast activation such as PDGF receptor, fibroblast growth factor receptor (FGFR), and vascular endothelial growth factor receptor (VEGFR) (Figure 4). In vitro and animal data support the anti-fibrotic effect of nintedanib in inhibiting crucial pathways in the initiation and progression of lung fibrosis. For instance, nintedanib inhibits the secretion of the pro-fibrotic cytokines IL-4, IL-5, and IL-13 in the peripheral blood of SSc patients. In addition, its anti-fibrotic effect is partly mediated by preventing the polarization towards the pro-fibrotic M2 macrophages [196]. Nintedanib may also exert its anti-fibrotic efficacy by restraining the migration and differentiation of fibroblasts and fibrocytes. Interestingly, in vitro data have shown that nintedanib downregulates the transition of fibrocytes towards myofibroblasts, and thus the pro-fibrotic effect of the latter is reduced [197,198]. Nintedanib has received FDA approval for the treatment of IPF. Since IPF shares pathogenic commonalities with SSc-ILD, the use of nintedanib to reduce lung fibrosis in patients with SSc-ILD has been recently evaluated in the SENSCIS trial [199]. This trial included 576 patients with SSc-ILD that were randomly assigned (1:1 ratio) to receive 150 mg of nintedanib two times daily or placebo for 52 weeks. Deterioration of lung function in the nintedanib arm was significantly lower compared to the control group. More specifically, the adjusted annual rate of decline in FVC was −52.4 mL in patients treated with nintedanib compared to −93.3 mL in the placebo group (*p* = 0.04). Based on the results of this study, in 2019, nintedanib was the first FDA-approved treatment for patients with SSc-ILD [200]. It is worth mentioning that no significant clinical benefit was observed in skin fibrosis measured by mRSS over a 1-year period. To our knowledge, no clinical data verifying the in vitro and in vivo anti-fibrotic mechanisms have been published to date. An open-label, extension trial, assessing the long-term safety and efficacy of nintedanib in 444 SSc-ILD patients, is ongoing and expected to provide data on prolonged nintedanib therapy and its exact mechanism of action in SSc patients (NCT03313180).

### 6.2. Tofacitinib

Except for the Ab1 and PDGF tyrosine kinases, inhibition of Janus kinases (JAK) is another potential treatment with anti-inflammatory properties. Recent data provide evidence that the JAK/STAT signaling pathway is highly activated in SSc skin biopsies [201]. Tofacitinib is a small molecule JAK inhibitor, with a structure similar to ATP, and has been used in the treatment of RA. Regarding its mechanism of action, it enters the cell with passive diffusion and binds to the ATP site of the JAK1 and JAK3 receptors. As a result, it inhibits the phosphorylation and activation of signal transducer and activator of transcription proteins (STATS), which leads to the downregulation of IL-6 expression [202] (Figure 4). In view of this mechanism, the use of tofacitinib in SSc might be of interest. The results from an observational study suggest tofacitinib as a potential treatment in reducing skin thickening of patients with refractory dcSSc [203]. More specifically, tofacitinib-treated patients showed a significantly shorter response time compared to the conventional immunosuppressive group, with a mean change of −3.7 (*p* = 0.001) in mRSS already 1 month post-treatment. After 6 months of treatment, the mean change in mRSS was even greater between the two groups (−10.0 tofacitinib vs. −4.1 conventional immunosuppressants, *p* = 0.026). Similarly, in another pilot study, patients treated with tofacitinib showed a significantly higher improvement in skin fibrosis compared to patients treated with MTX [204]. These results indicate that tofacitinib might be even more effective in reducing fibrosis than conventional immunosuppressants, further showing a quicker and higher response rate. The tolerability and efficacy of tofacitinib have been recently evaluated in a small clinical phase I/II trial in 15 SSc patients. According to the preliminary results, tofacitinib is well tolerated and shows a trend towards improving fibrosis (NCT03274076). Further evaluation of tofacitinib in dcSSc seems warranted.

**Table 5:** Clinical trials evaluating the efficacy of tyrosine kinase inhibitors in SSc treatment.

Drug	Target	Type of Trial(s)	Duration (Months)	Patients	Results
Imatinib mesylate	Inhibits PDGFR and TGF-β signaling	Multicenter, open-label RCT [193]	6	27 dcSSc	Mean decrease in mRSS was 21% compared to baseline (*p* < 0.001)
Nilotinib	Same as imatinib, but 20–30-fold more potent	Open-label, single-arm trial [195]	8	10 early dcSSc	Promising reduction of 23% in mRSS 12 months post-treatment (*p* = 0.01)
Nintedanib	Inhibits PDGFR, FGFR, and VEGFR signaling	Double-blind RCT (SENSCIS) [199]	13	576 SSc-ILD	Adjusted annual rate of decline in FVC −52.4 mL compared to −93.3 mL in the placebo group (*p* = 0.04)
Tofacitinib	Inhibits JAK/STAT signaling	Double-blind RCT (NCT03274076)	6	15 dcSSc	Preliminary data show a trend towards improved fibrosis

## 7. Conclusions and Future Perspectives

SSc is a disabling, chronic, auto-immune disease accompanied by high mortality and morbidity rates. In this review, we examined the potential effects of anti-inflammatory and immunosuppressive treatments in attenuating fibrosis. To do this, we evaluated the first-line, generalized as well as cell- and cytokine-specific anti-inflammatory and anti-fibrotic treatments, including the main therapies that have been used or are currently being tested in clinical trials.

To begin, among the broad-spectrum treatments, MTX has been well tolerated, but its efficacy in reducing fibrosis is still controversial. Although CYC’s use seems efficient in reducing fibrosis, its high toxicity limits its use, and it has now been replaced with MMF. MMF is well tolerated, but its use has not been fully examined. On the other hand, cellular targeting of inflammation with molecules that reduce the number of B or T cells, such as rituximab, belimumab, and abatacept, has exhibited a potential effect in diminishing fibrosis compared to broad-spectrum immunosuppressants. Furthermore, the cytokine signaling-specific antibodies rilonacept, basiliximab, fresolimumab, and metelimumab show a trend towards reducing fibrosis, but the lack of large phase III trials limits their potential addition to SSc treatment guidelines. Among the TK inhibitors, nintedanib and tofacitinib are two promising therapies in halting SSc-related fibrosis. Based on encouraging outcomes from large phase III RCTs, tocilizumab and nintedanib have been FDA approved for the treatment of SSc-ILD. On the positive side, ASCT shows increased event-free and improved overall survival rates, but its use is limited to younger patients with early dcSSc that fulfill a list of very strict inclusion criteria. Careful patient selection is vital in reducing the relatively high mortality rates that accompany ASCT.

In conclusion, we are in a new era of a multitude of clinical trials with drugs targeting specific pathogenic cells and biological pathways related to SSc. Several anti-inflammatory treatments have been used or tested in SSc patients, but only a mild to moderate improvement in reducing fibrosis has been demonstrated. However, the level of published evidence on the effectiveness of each tested drug varies greatly among case series and observational studies, and only a few RCTs have been conducted [205]. Thus, conclusions and comparisons between the efficacy of different drugs should be handled with caution. The anti-inflammatory approaches described show a trend towards reducing fibrosis, but the effect is moderate and, in many cases, controversial. The lack of complete understanding of the pathophysiology and the rare frequency of the disease are two obvious arguments that support this conclusion. Furthermore, SSc is a very complex and heterogeneous disease, and the traditional classification of the patients into the limited or diffuse form based on the severity of skin involvement is an oversimplification. Lately, multiple studies have utilized intrinsic gene expression analyses to classify SSc patients into four categories: the inflammatory, fibroproliferative, limited, and normal-like subsets [206]. It is currently not certain if these categories are reflective of stable disease states that differ between patients. Other studies suggest that they concern different stages of the SSc disease process [80]. Various disease manifestations of SSc, such as ILD, pulmonary hypertension, and gastrointestinal disease, develop with a time course that differs from that of the skin manifestations. The underlying maladaptive innate and adaptive immune responses likely differ between these pathologies. Taken together, we believe that in-depth insight and measurement tools of the pathological processes may yield markers to determine the biological processes driving specific disease manifestations. Such markers will help to determine the best treatment approach in individual cases.

Furthermore, targeted drug selection is expected to show more remarkable results in the anti-inflammatory therapies that have been mentioned. Additionally, to understand if new drugs are effective, there is also a need for a better understanding of the pathological processes driving disease features. The use of novel and advanced molecular tools such as single-cell RNA sequencing is constantly advancing our knowledge about novel pathogenic cytokines, antibodies, and genes implicated in the pathogenesis of SSc. This knowledge will facilitate more personalized treatments. For example, scleroderma patients that exhibit high amounts of the Th2 cytokines IL-4 and IL-13 early after diagnosis, or those with prominent ILD, could be treated with immunotoxins or monoclonal antibodies that have been designed to block the IL-4 or IL-13 pathway and have shown promising results in anti-tumor, IPF, and asthma treatment. Another suggestion for future studies would be to evaluate the outcome of combined biological therapies.

## Data Availability

Not applicable. No new data were generated or analyzed in this study.
